# Establishing a new methodology for annelid studies: a biometric study of the ragworm *Hediste diversicolor* (Müller, 1776)

**DOI:** 10.7717/peerj.20736

**Published:** 2026-02-03

**Authors:** João Almeida, Carlos Antunes, Bruno Spacek Godoy, Dimitri de Araujo Costa

**Affiliations:** 1 FCUP—Faculty of Sciences, University of Porto, Porto, Porto, Portugal; 2 CIIMAR—Interdisciplinary Centre of Marine and Environmental Research, University of Porto, Matosinhos, Porto, Portugal; 3 Aquamuseu do Rio Minho, Vila Nova de Cerveira, Viana do Castelo, Portugal; 4 INEAF—Instituto Amazônico de Agriculturas Familiares, Universidade Federal do Pará, Belém, Pará, Brazil; 5 NEAP—Núcleo de Ecologia Aquática e Pesca da Amazônia, PPGEAP—Programa de Pós-Graduação em Ecologia Aquática e Pesca, Universidade Federal do Pará, Belém, Pará, Brazil; 6 GIBI—Grupo de Investigação Biológica Integrada, CEABIO—Centro de Estudos Avançados da Biodiversidade, Universidade Federal do Pará, Belém, Pará, Brazil

**Keywords:** Nereididae, Polychaeta, Chaetiger, Developmental biology, Morphometry, Life cycle

## Abstract

In this study, we propose a new biometric methodology for annelids, based on the number of chaetiger segments as an indicator of growth (independent variable), relating to other structures, such as the number of paragnaths and parapodia, using the commercial species *Hediste diversicolor* (Müller, 1776) as a model. This species belongs to the family Nereididae, found in estuarine environments along the European Atlantic coast (DOI 10.11646/zootaxa.5696.1.2). This species has several appendages with sensory functions along the body that help the animal navigate in its environment. It also has specialised feeding structures in the peristomium that are essential for taxonomic identification, called paragnaths. The parapodium has distinct chaetal arrangements that distinguish it from other species in the same genus. Due to the importance of economic activities such as fishing and aquaculture, this ragworm is a typical protagonist for studies in multiple areas, with a well-documented life cycle. However, knowledge is lacking on the growth and evolution of individual structures and appendages of the body in this species during its life cycle. Our findings revealed a significant positive correlation between the number of chaetiger segments and most of the morphological structures analysed, evidencing a proportional growth of most of these features, related directly to the chaetigers segments. Simple chaetae, a specific type of chaeta only present in the genus *Hediste*, were only found in individuals with more than 25 chaetiger segments, possibly indicating a functional change as the worm matures. Simple chaetae tend to be found earlier along the body axis, closer to the median chaetiger as the organism matures. The results illustrate how *H. diversicolor* develops over time, highlighting the developmental processes and representing the first biometric study of nereidid polychaetes, based on chaetiger count as a proxy for biometric growth, to define new possible life cycle stages, for supporting aquaculture purposes and other research fields.

## Introduction

Polychaetes are a diverse group of annelid worms, many of which bear bristle-like structures called chaetae. The anterior region of polychaetes comprises the peristomium, which houses the mouth and various sensory structures, and the prostomium, which contains numerous sensory organs and structures that contribute to the characterisation of different families. Polychaetes can be divided into two main clades: sedentary and errant (Magalhães et al., 2021). Errant polychaetes, which are more mobile, typically have a more developed cephalisation process with well-defined eyes and antennae, while sedentary polychaetes have a reduced or absent cephalisation process (Viéitez et al., 2004).

The family Nereididae, Blainville, 1818, is a prominent group of errant polychaetes. Members of this family have a diamond-shaped prostomium with rounded corners that fuses with the peristomium (Fauchald & Rouse, 1997). The prostomium includes two pairs of eyespots arranged in a trapezoid shape, with the posterior pair positioned closer to each other. Typically, these polychaetes have two antennae, a pair of conical, bi-articulate palps, and small, short ciliated grooves, known as nuchal organs, located posteriorly to the eyespots. The peristomium usually carries four pairs of cylindrical tentacular cirri, but this number varies depending on the taxa. Still in the peristomium, the mouth has an opening on the ventral side. The eversible muscular proboscis or pharynx, which has chitinous jaws and two distinct rings when everted, is essential for feeding and is used to differentiate taxonomic groups within the family, based on the arrangement of the papillae or paragnaths (Fauchald & Rouse, 1997; Brusca, Giribet & Moore, 2022). The positions of paragnaths in said rings are extremely important for the taxonomy within the nereidids. These areas were standardised by Hjalmar Kinberg in 1865 to evaluate the distribution of the papillae or paragnaths on these rings (Bakken, Glasby & Wilson, 2009). A total of eight different areas are distinguishable, numbered with roman numerals (I, II, III, IV, V, VI, VII and VIII). The areas I, IIa/b, III and IVa/b are located in the maxillary ring and V, VIa/b, VII and VIII are located in the oral ring. These areas help to differentiate genera within the family Nereididae (Viéitez et al., 2004).

The genus *Hediste* shares many characteristics with the genera *Nereis* and *Neanthes*, such as the presence of conical paragnaths on both rings of the proboscis, and specific forms of parapodia and types of chaetae. A distinctive feature of *Hediste* is the presence of simple chaetae above the neuropodial acicula on posterior parapodia, along with other types of chaetae such as homogomph spiniger, heterogomph spiniger, and heterogomph falciger.

**Figure 1:**
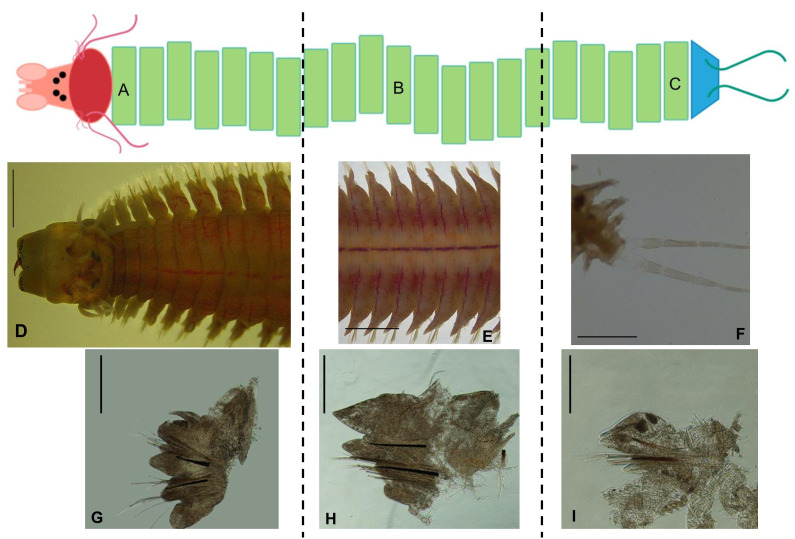
Simplified scheme of *Hediste diversicolor* and the appearance of different parapodia along the body. Pink: prostomium; red: peristomium; green: chaetiger segments; blue: pygidium; and the appearance of different parapodia along the body axis: (A, D) anterior region of the body; (B, E) median region of the body; (C, F) posterior region of the body; (G) first parapodium; (H) a median parapodium; (I) a posterior parapodium. Scales: (D–F) 2.0 mm; (G–I) 0.5 mm. Photos: Dimítri Costa.

**Figure 2:**
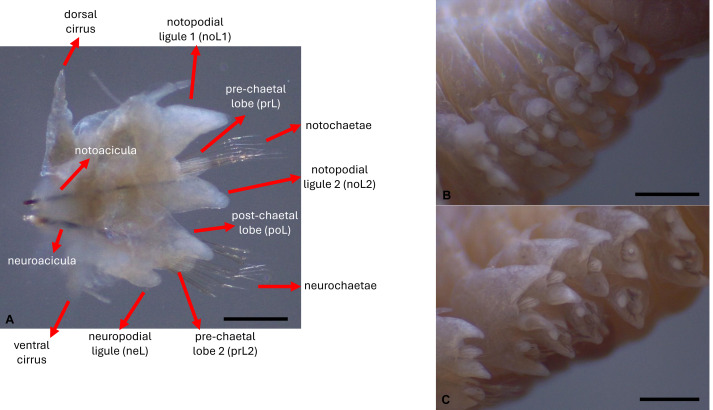
Parapodial features. (A) Cirri, parapodial lobes, chaetae and aciculae, (B) lateral view of anterior parapodia, and (C) median parapodia. Scales: (A) 2.0 mm; (B, C) 1.0 mm. Photos: Dimítri Costa.

The species *Hediste diversicolor* (Müller, 1776)—a commercial polychaete widely used for aquaculture purposes, can be found along the European Atlantic coast, Baltic Sea, Mediterranean Sea and North Africa (Teixeira et al., 2022)—in estuarine and brackish areas (Costa et al., 2025). Its body can reach up to 10 cm in length and six mm in width, and is thick-bodied, dorsoventrally flattened and tapering towards the posterior end, comprising an average of 90–95 chaetiger segments in fully grown specimens (Viéitez et al., 2004). The prostomium has two short antennae between a pair of palps that are tapered and biarticulated, with the anterior region called the palpostyle, wider than long, which is smaller than the posterior region named palpophore. It has two pairs of eyespots set up in a trapezoid shape, with the posterior pair being closer together than the anterior pair. The peristomium is approximately the same length as the rest of the chaetiger segments. There are four pairs of tentacular cirri formed from the peristomium, with cirrophore wider and shorter than cirrostyle. The most posterior cirri pair is wider and longer than the rest. The proboscis has a pair of brown jaws with teeth (serrated). Conical paragnaths can be found on both proboscis rings, with no paragnaths on area V. The chaetiger segments have bilobated parapodia with slight differences in shape and length along the body (Figs. 1A–1I). The notopodium in the first parapodia is composed of notopodial cirri and a notopodial ligula, without aciculae or chaetae (Fig. 1G). From the third chaetiger onwards (Figs. 1A–1I), the notopodium has an acicula, two notopodial ligules, one above and one below the acicula and a pre-chaetal lobule, with homogomph spiniger chaetae. The neuropodium has an acicula, and it is composed of the neuropodial cirrus, a pre-chaetal and a post-chaetal lobule and a neuropodial ligule (Fig. 2A). Supra-acicular chaetae are from the homogomph spiniger and heterogomph falciger type, although on the posterior chaetiger segments, simple chaetae can appear. Infra-acicular chaetae are from the heterogomph spiniger and falciger, even though homogomph falciger can rarely appear. The neuropodium tends to become smaller compared to the notopodium towards the more posterior segments (Figs. 1G–1I, 2B–2C). The last segment is an achaetiger that contains the pygidium, composed of a pair of long anal cirri and the anal orifice.

This species reproduces only once in a lifetime, with individuals dying shortly after reproduction. They use up to 70% of their total energy in the production of gametes inside the celoma, causing an increase in body mass and a stop to their growth (Fidalgo-e-Costa, 2003), due to the incubation of gametes. Sexual maturation is induced by an increase in temperature, and spawning is influenced by the lunar cycle (Dales, 1950). Sexual maturation is reached at one to two years. Both male and female *H. diversicolor* are indistinguishable before maturation, presenting a reddish-brown colour. After maturation, males acquire a light green colour and females a darker green (Jamieson, 2006); this difference is explained by the increase of white sperm in the celoma of males (Scaps, 2002).

Unlike other nereidids, *H. diversicolor* lacks an epitoke stage or planktonic larval stage (swimming stage), having a fully benthonic life cycle. Mature females lay their eggs inside galleries, and mature males lay sperm at the entrance of the galleries. Then females increase their ventilatory activity inside the galleries, leading sperm into the eggs and increasing the chance of fertilisation (Scaps, 2002). Fertilised eggs develop quickly inside the galleries, still protected by the females until they die. Larvae hatch with three chaetiger segments at the trochophore stage and develop (first chaetae appear; sense organs like eyes, palps, antennae, dorsal and anal cirrus appear; formation of the jaws; growth in length) while feeding on mucus and bacterial particles inside the gallery. At 10 to 14 days post-hatching, larvae have six to eight chaetiger segments (Bartels-Hardege & Zeeck, 1990; Wang et al., 2020) and are ready to leave the parental galleries (Bartels-Hardege & Zeeck, 1990; Scaps, 2002; Hesselberg, 2006). It is at this developmental stage that they start showing burrowing behaviour, carving their own burrows in the substrate (Scaps, 2002).

This annelid is an important part of the trophic chain, serving as prey for various organisms and contributing to the recycling of organic matter in its habitat (Fauchald & Jumars, 1979; Dierschke et al., 1999; Jumars, Dorgan & Lindsay, 2015). Additionally, *H. diversicolor* is used as bait in fishing, impacting geographic communities through the dissemination of different populations (Fidalgo-e-Costa, Passos & Cancela da Fonseca, 2003; Font, Gil & Lloret, 2018).

While progress has been made in understanding the nervous systems and sense organs of mature annelids, studies concerning the development of these structures throughout the life cycle remain limited. Research in this field is crucial for evolutionary development studies and for assessing environmental impacts on organism development (Winchell, Valencia & Jacobs, 2010; Pires et al., 2016).

Despite being well-known invertebrate taxa, biometric studies represent a gap in polychaete research, including for commercial species, like *H. diversicolor*, demonstrating the importance of this study to a set of other research areas—aquaculture, biology, and others.

In this way, this study is the first biometric morphological characterisation of *H. diversicolor*, by examining structures across animals at different developmental stages using chaetiger count as a proxy for biometric growth. We seek to explore and identify morphological changes and potential differences that improve our knowledge of their growth and development.

## Material and Methods

### Study area

**Figure 3:**
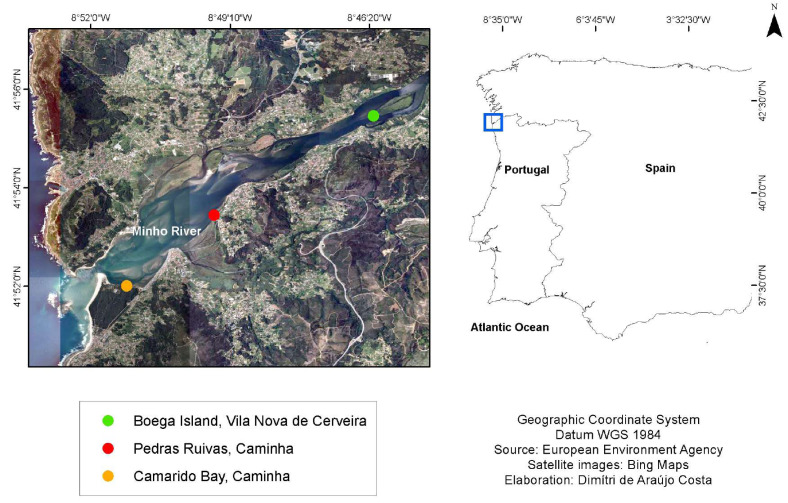
Study area in Minho River. Study area in the international section of Minho River (Southern Europe), displaying the points selected for sampling.

The study area chosen was the Minho River estuary, located on the northwest coast of the Iberian Peninsula (Southern Europe). The Minho River creates a natural border between Portugal and Spain for an 80 km downstream section before flowing into the Atlantic Ocean. This estuary has a maximum width of approximately two km next to the mouth. Tidal influence extends approximately 40 km upstream, comprising a total estuarine area of 23 km^2^. The estuary is mesotidal, characterised by a predominantly semidiurnal tidal regime, with a variation between two m at low tides and four m at spring tides, and its maximum depth is 11 m below the average sea level (Díaz, Rodrigues & Guedes Soares, 2020). Sampling sites were selected along the Minho River estuary according to the already-known existence of *H. diversicolor* in the area. Thus, three sites were chosen, due to previous sampling which showed that there are places along the river with large numbers of these annelids: (1) Camarido Bay, Caminha (41°52″01.2″N 8°51″00.3″W); (2) Pedras Ruivas village (41°53″28.4″N 8°49″30.8″W); (3) and Boega Island, Vila Nova de Cerveira (41°55″28.2″N 8°46″13.8″W) (Fig. 3). These sites were selected solely for the purpose of collecting specimens, with no specifications for comparison tests between sites.

**Figure 4:**
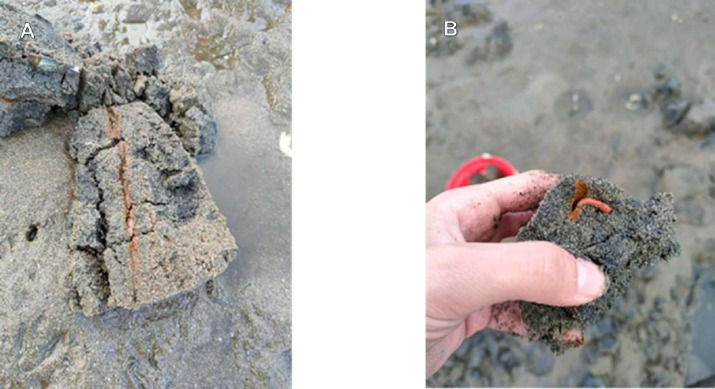
Bioturbation on the sediment. (A) Transversal section of a borrow inside the sediment, (B) *Hediste diversicolor* exiting the borrow through the surface opening. Photos: João Almeida.

The sediment at these sites consisted of a mixture of sand and mud with a few large rocks. The exact point for the collection of sediment samples was selected upon the identification of bioturbation in the sediment that is typical of this species (Figs. 4A–4B).

### Sample collection

Sediment samples were collected using shovels and placed in buckets to ease transportation. Immediately after arrival at the laboratory, the samples were sorted to protect the organisms from desiccation stress. To ease the collection of the polychaetes, small amounts of sediment were placed in plastic trays where water with the same salinity as the sampling site was added. Annelids were placed in beakers within water samples brought from the Minho River estuary. Specimens were selected based on the number of chaetiger segments in order to ensure a uniform distribution—according to our initial hypothesis that this variable could influence the growth pattern of the annelid morphological characteristics. The largest animals collected in this initial stage were then preserved in 70% alcohol for further biometric analysis. The rest was sent to a purposely build monoculture tank (see the description below), which served as a support for obtaining organisms at various stages of development to finish all measurements. All annelids were fixed in alcohol before the biometric analysis of this study was carried out.

### Species identification

Morphological identification was carried out using dichotomous identification keys from relevant literature to guarantee that all the worms analysed belonged to the target species (Viéitez et al., 2004). Photographs taken using a stereo microscope (Leica EZ4W; Leica, Wetzlar, Germany, software basic version) and drawings were made to document the essential taxonomic characteristics of *H. diversicolor*.

### Monoculture tank setup

**Figure 5:**
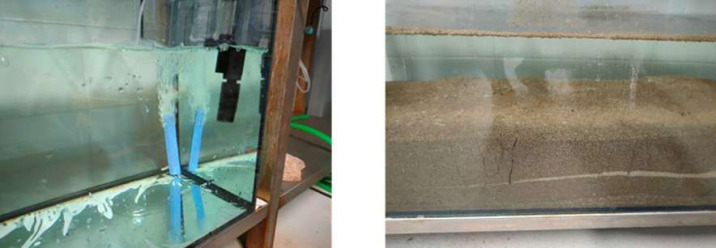
Monoculture tank setup. (A) Tank equipped with aerator and filtration pump filled with a brackish water solution, (B) tank with sediment and polychaetes, being possible to observe bioturbation formed by the annelids in the sediment. Photos: João Almeida.

A monoculture tank with a capacity of 200 litres was set up to hold specimens at different stages of development collected from the river. A brackish water solution (salinity 22–24) was placed in the tank. To keep oxygen at adequate levels and the water clean, the tank was equipped with an aeration pump and a filtration system (Fig. 5A). After the sediment from one of the sampling sites had been carefully examined and cleaned of competing species, it was used as a substrate for the tank (Fig. 5B). The polychaetes were fed once a day with SERA^®^ Pond Flakes Nature. The density of polychaetes in the tank was not controlled during the experiment, being possible for the animals to reproduce in the tank. A multiparameter probe was used to monitor water quality and some parameters such as pH, temperature, conductivity, dissolved oxygen, and salinity. Whenever possible, the salinity was adjusted, adding salt water or adding fresh water conform needed to keep it in a range close to the observed in the Minho River estuary.

### Biometric and anatomical analysis

**Figure 6:**
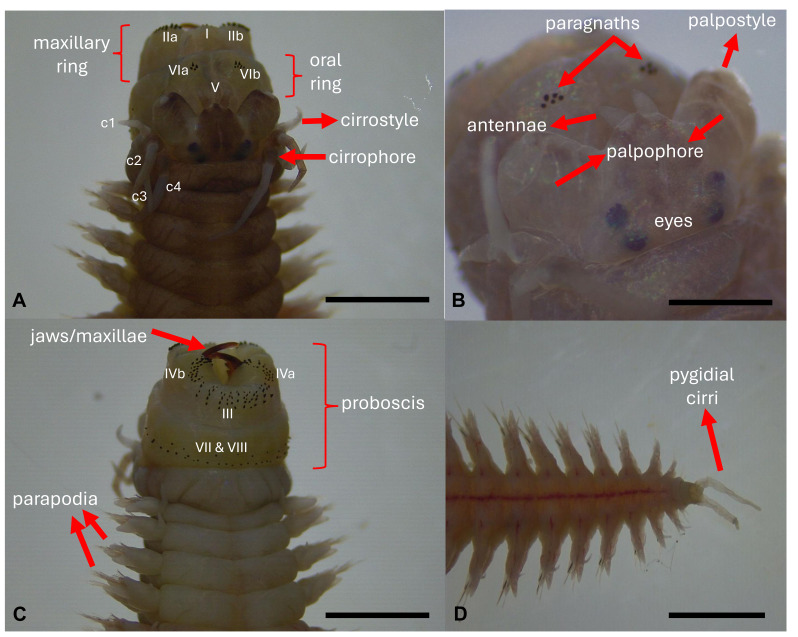
Main morphological features of *Hediste diversicolor*, as a basis for the biometric analysis in this study. (A) Dorsal view, which c1 represents first pair of cirri (c2, the second one, and so on), and the Roman numerals represent each area of the proboscis, (B) highlight of the prostomium (head), (C) ventral view, (D) highlight of the posterior end (pygidium). Scales: (A, C, D) 2.0 mm; (B) 1.0 mm. Photos: Dimítri Costa.

Biometric analyses were conducted according to the standard descriptive methodology for the family Nereididae (Viéitez et al., 2004) using an optical microscope (Nikon SMZ800; Nikon, Tokyo, Japan) and a stereo microscope (Leica EZ4W). Measurements were performed using ImageJ software. Each specimen was measured, the number of chaetigers counted, as well as the number of paragnaths (pgn) and their relative position in the eversible proboscis noted (Figs. 6A–6C). The maxillae (Fig. 6C) were also measured in width and length, and the number of teeth in each jaw was counted. These structures were counted and measured due to their importance as taxonomic characteristics. The prostomial palps were divided into palpophore and palpostyle (Fig. 6B), and each of these sections was measured for width and length. The width and length of the lateral antennae were measured as well. In the peristomium, each cirrus was divided into cirrophore and cirrostyle (Fig. 6A), and the width and length of each segment were registered. A detailed anatomical analysis of parapodia was performed (Fig. 2A). A set of seven parapodia were chosen according to their relative position (Figs. 2B–2C), these being the first anterior parapodium; a parapodium in the transition zone between anterior parapodia and median parapodia; a median parapodium located at a distance of the same number of chaetiger segments between the anterior-median transition parapodium and the median-posterior transition parapodium; a median-posterior parapodium; a posterior parapodium; the parapodium anterior to the first parapodium that carries simple chaeta; and the first parapodium with simple chaeta. Measurements included the width and length of notopodial and neuropodial cirri, notopodial ligule 1 (noL1), pre-chaetal lobe (prL), notopodial ligule 2 (noL2), post-chaetal lobe (poL), pre-chaetal lobe 2 (prL2) and neuropodial ligule (neL) (Fig. 2A).

The length of the notopodial and neuropodial aciculae was noted when present. The types of chaetae in each parapodial region (notopodial, neuropodial, supra-acicular, and infra-acicular) were noted. If the animals analysed had suffered any trauma prior to the analysis, or if the animals had a low number of chaetiger segments, then only the anterior, median and posterior parapodia were sampled. If the animals did not have simple chaetae, these parapodia were not sampled either. Finally, the width and length of the pygidium cirri (Fig. 6D) were measured, including the cirri segment lengths. A total of 30 specimens of *H. diversicolor* were biometrically analysed. Due to the size of the different animals, certain characteristics were very difficult to count, such as the number of paragnaths, as these become more markedly coloured during the development of the organism. Other structures, such as the parapodia, become difficult to differentiate visually when the polychaetes are at an earlier stage of maturation. Some of the animals analysed appeared to have suffered trauma before analysis, which is why certain structures were sometimes absent. The number of organisms analysed, therefore, differs according to the target structure analysed.

### Statistics analysis

Data were organized and analysed using Microsoft Excel™ and R software v.4.3.1 (R Core Team, 2023). To reduce the dimensionality of the dataset and avoid the performance of multiple correlated univariate tests, we applied Principal Component Analysis (PCA) to groups of morphologically and functionally related structures: (1) palps, (2) proboscis paragnaths, (3) peristomial cirri, (4) anterior parapodia, and (5) median-posterior parapodia. For each group, we used the threshold of 70% of explanation of variance for the number of principal components (PC1) extracted and correlated with the number of chaetiger segments using Pearson’s correlation. For structures not suitable for PCA groups (antennae, jaw teeth, transitional parapodia, and pygidial cirri), univariate Pearson correlations were performed between each morphological measurement and the chaetiger number by using PAST software, v.4.13 (Hammer, Harper & Ryan, 2001). Multivariate analyses were performed using the vegan and ggplot2 packages in R. Shapiro-Wilk and Anderson-Darling Normality tests were performed to check for normal distribution, considering a significance value of *p* ≤ 0.05. Levene’s homoscedasticity was evaluated by creating a linear regression between the number of chaetiger segments and the measures obtained from each structure in order to obtain fitted values and residuals. With these values, an XY scatter plot was created to determine if the variance was constant. Based on the normal data distribution (*W* = 0.8901–0.9595; *p* = 0.3837–0.6845) and homoscedasticity’s residual variances, there was no significant difference (*F* = 0.1001–0.1173; *p* = 0.6998–0.7346).

In this way, our initial hypothesis is that the number of chaetigers segments is an effective variable for correlating growth patterns with the main morphological characteristics of *H. diversicolor*, as it can also be applied to all annelids.

## Results

**Figure 7:**
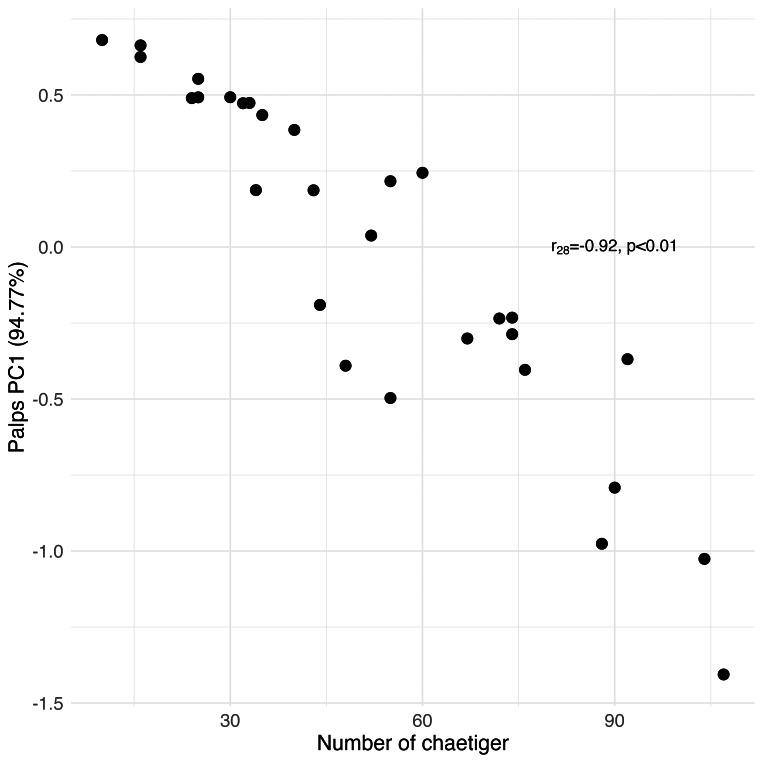
Principal Component Analysis between the length and width of the palps and the number of chaetiger segments.

**Figure 8:**
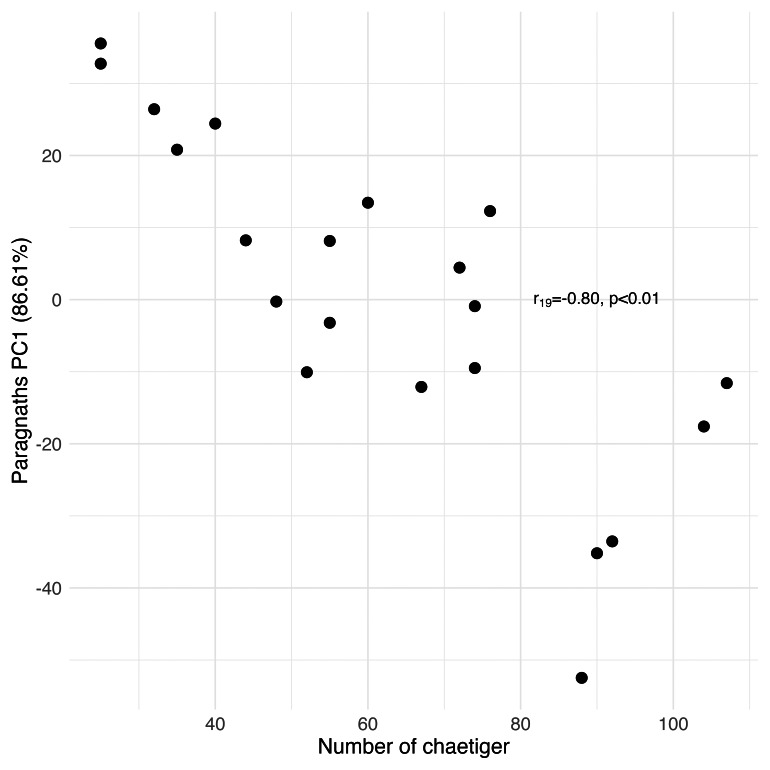
Principal Component Analysis between the number of proboscis paragnaths with the number of chaetiger segments.

**Figure 9:**
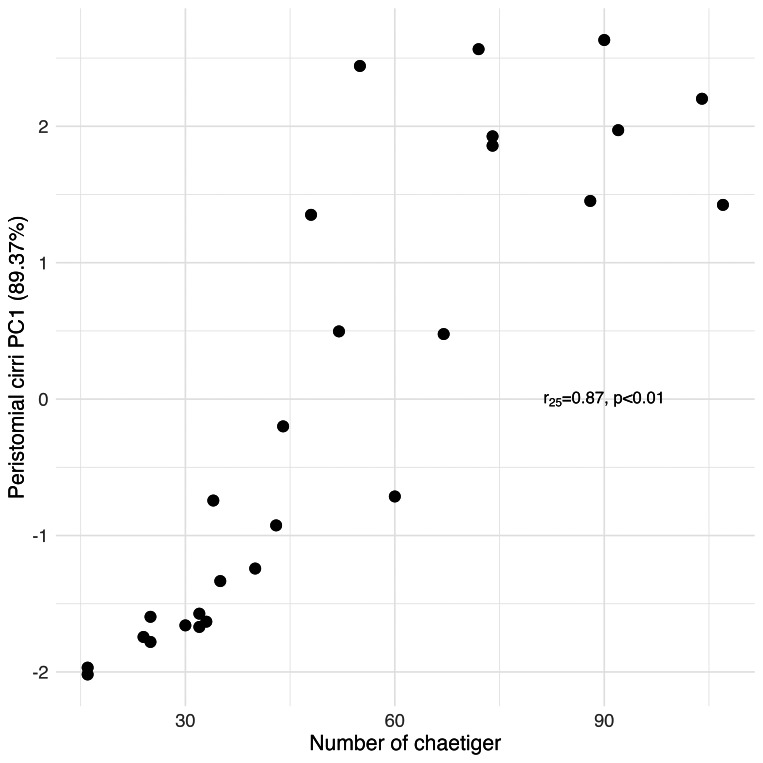
Principal Component Analysis between the length and width of the structures from the peristomial cirri and the number of chaetiger segments.

**Figure 10:**
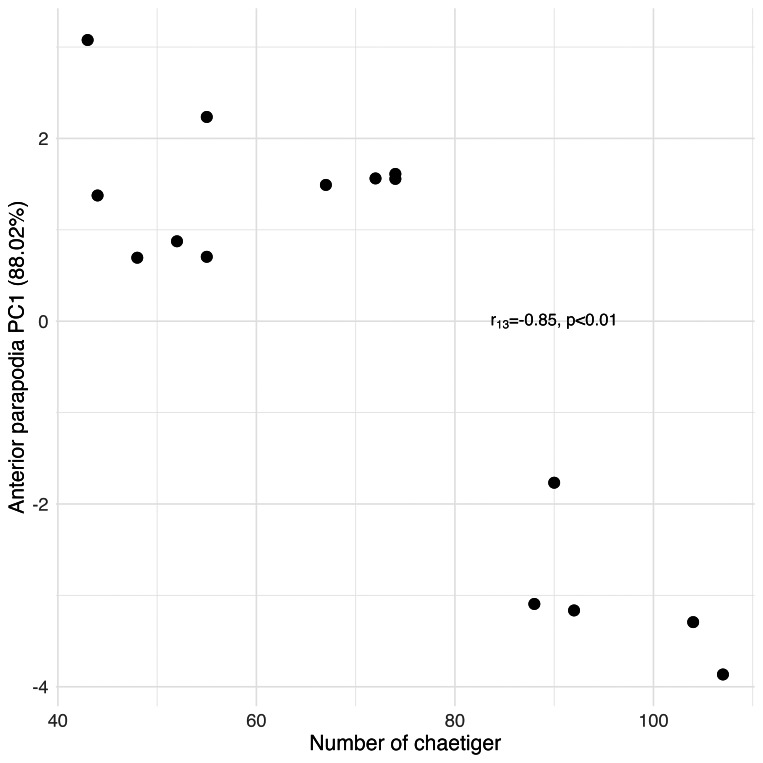
Principal Component Analysis between the anterior parapodial structures and the number of chaetiger segments.

**Figure 11:**
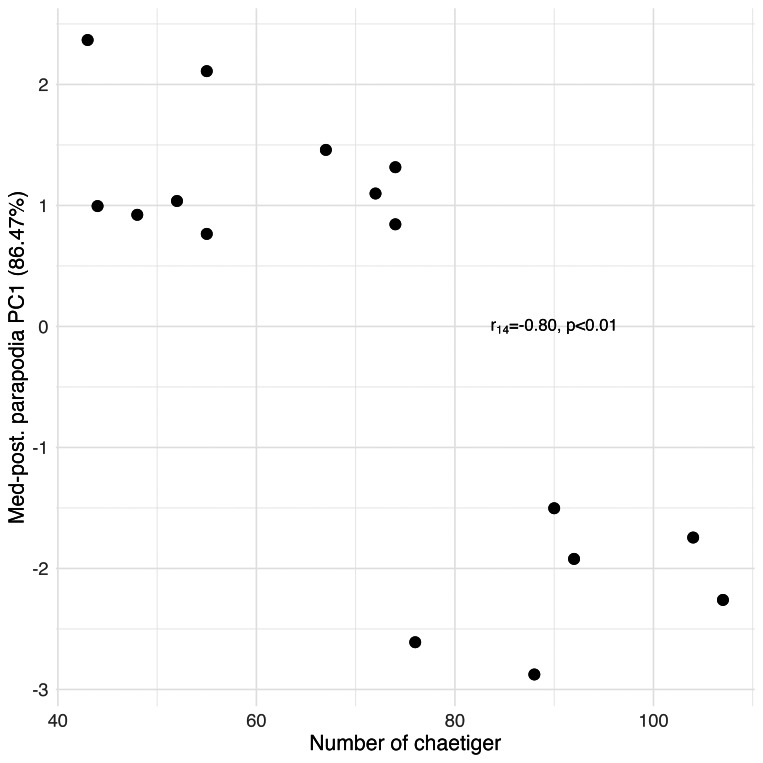
Principal Component Analysis between the median-posterior parapodial structures and the number of chaetiger segments.

Principal Component Analysis revealed that the first principal component (PC1) explained the majority of variance in each morphological group: palps (94.77%), proboscis paragnaths (86.61%), peristomial cirri (89.37%), anterior parapodia (88.02%), and median-posterior parapodia (86.47%). All the PC’s were related to several characters of the morphological group (see Table S1). The PC1 scores for all groups showed significant correlations with the number of chaetiger segments (Pearson correlation, *p* < 0.01), indicating a strong overall allometric growth pattern across all measured structures (Figs. 7, 8, 9, 10 and 11).

**Figure 12:**
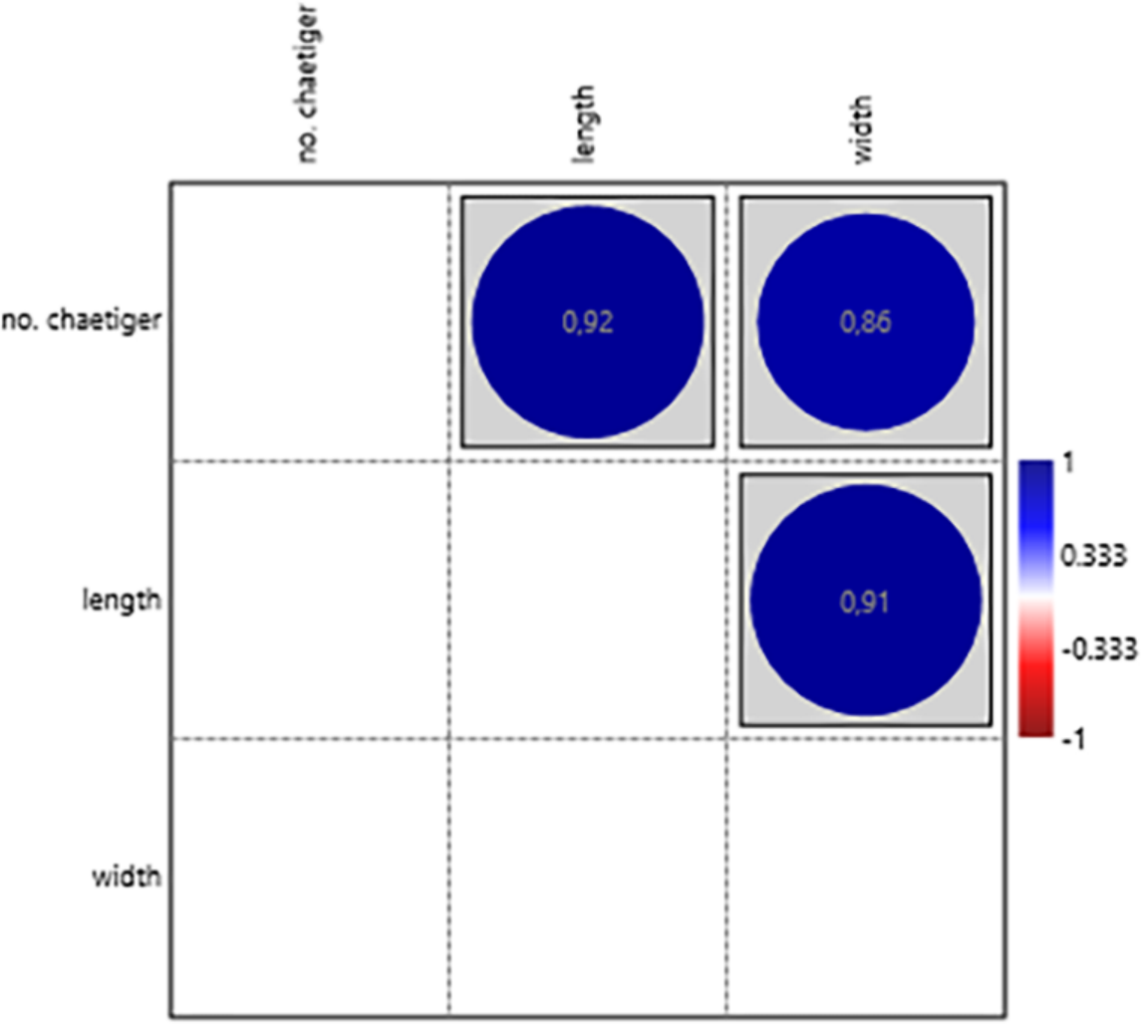
Correlation between the length and width of the lateral antennae and the number of chaetiger segments.

**Figure 13:**
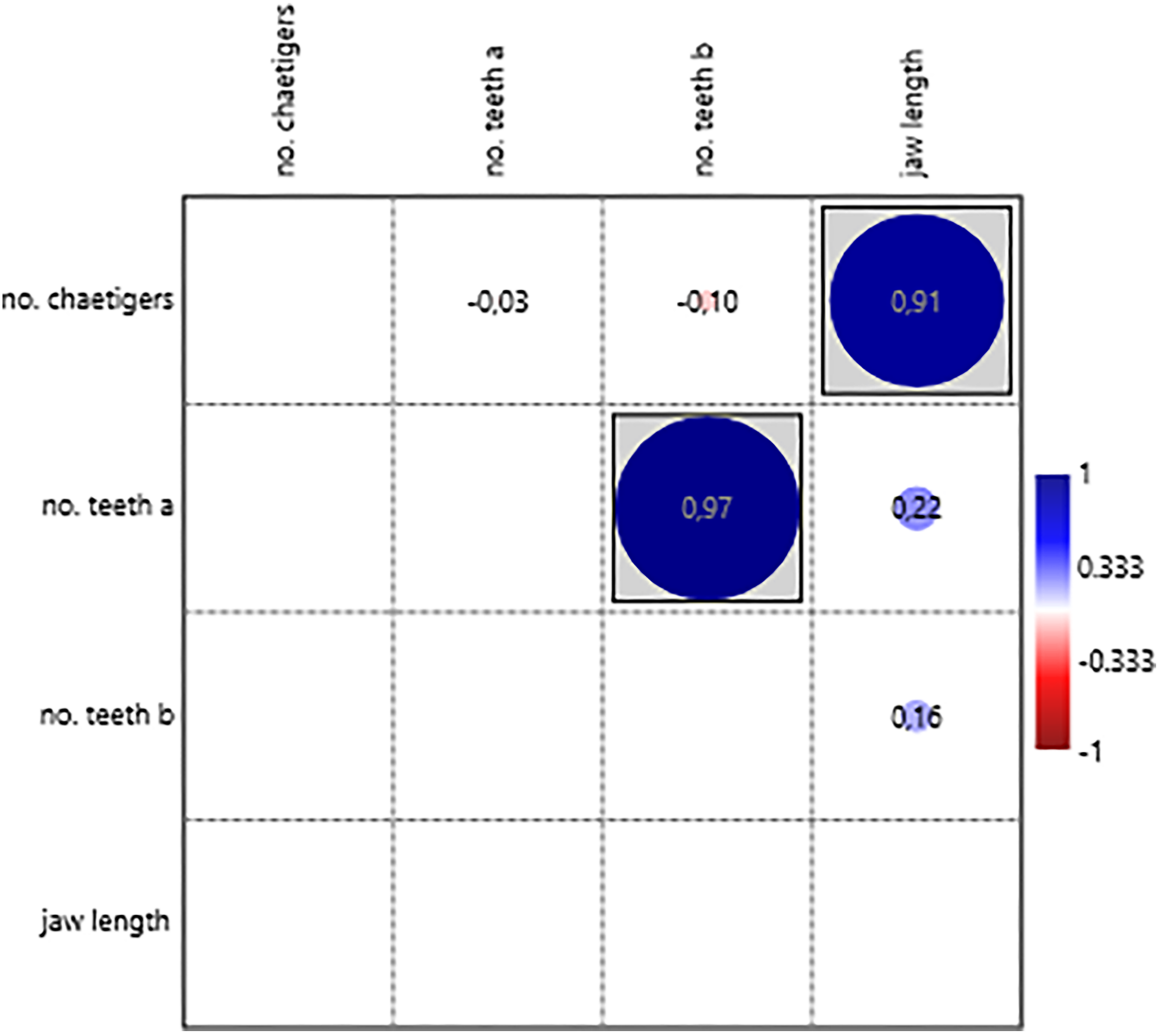
Correlation between the number of teeth from the right and left, and length of the jaws with the number of chaetiger segments.

**Figure 14:**
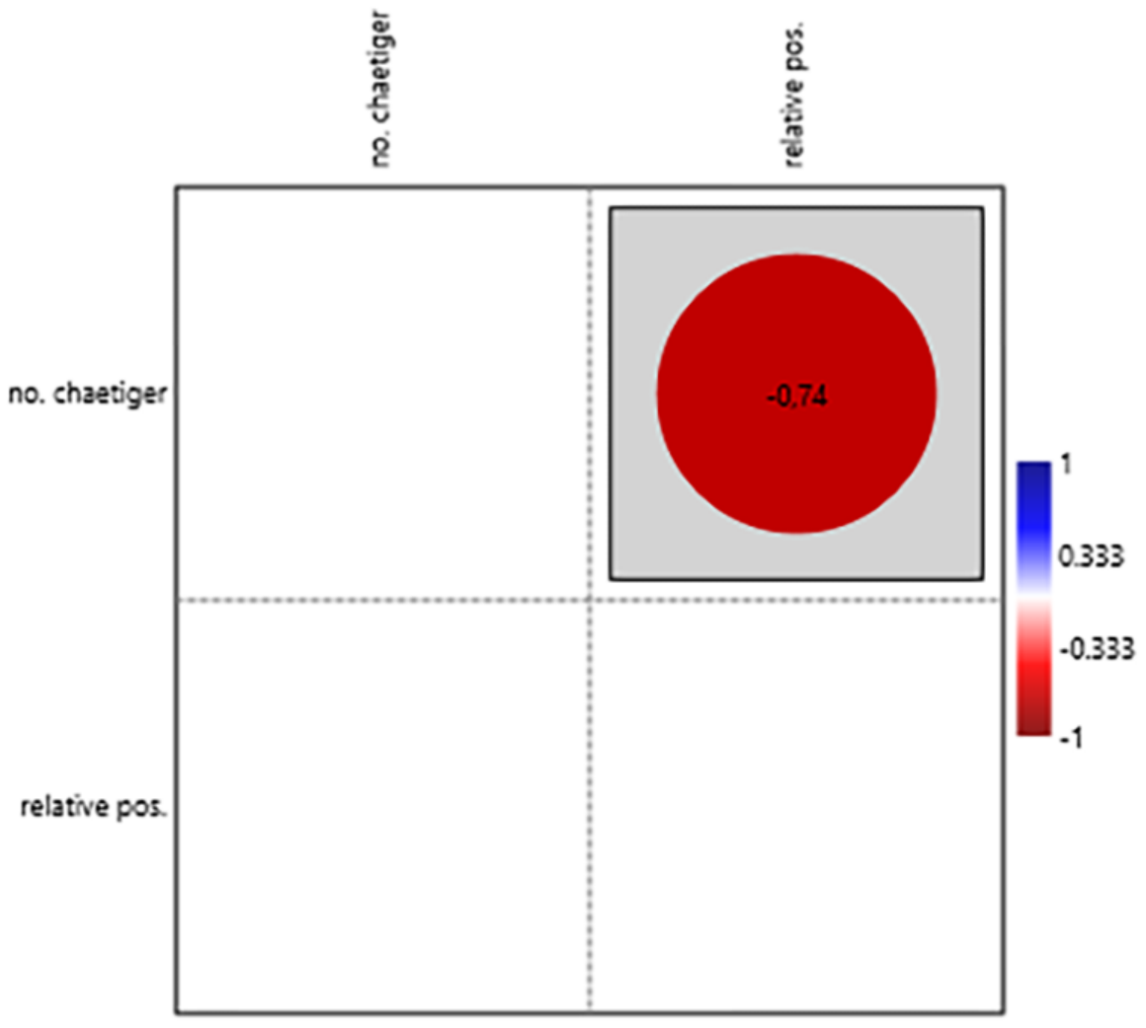
Correlation between the relative positions of the first chaetiger segment with simple chaetae and the number of chaetiger segments.

**Figure 15:**
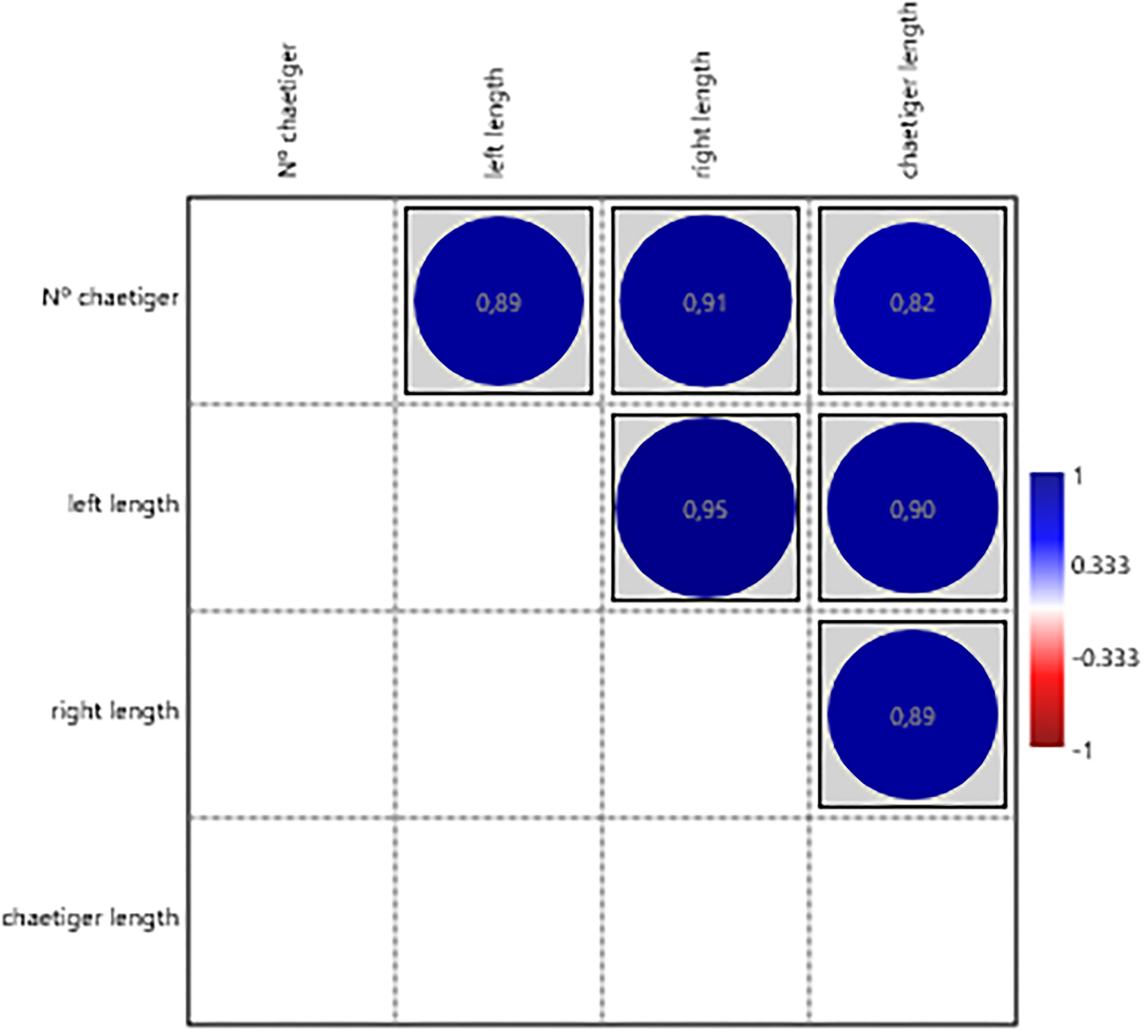
Correlation between the length of the right and left pygidial cirri and their sections and the number of chaetiger segments.

For structures analysed individually, significant positive correlations were found between chaetiger number and prostomial structures (length and width of lateral antennae, Fig. 12); jaw structures (length of jaws Fig. 13); simple chaetae (Fig. 14); and pygidial structures (length of pygidial cirri and their segments Fig. 15). The relative position of the first parapodium with simple chaeta showed a significant negative correlation with chaetiger number, indicating these structures appear earlier along the body axis as the worm matures (Fig. 14).

Data corresponding to individual growth rates of each measure from the prostomial, peristomial, parapodia and cirri per each new chaetiger segment are available in Tables 1–4.

All the data gathered from the quantification and measurement of the *H. diversicolor* structures can be seen in the supplementary material tables (Tables S2–S7).

These results confirm that the number of chaetiger segments is a robust proxy for overall morphological development in *H. diversicolor*, with most structures showing coordinated growth along a common size axis.

## Discussion

From the analysis of correlations between the various target structures with different functions, such as sensory or locomotor, and the number of chaetiger segments, important data were obtained to understand the morphological development of *Hediste diversicolor*. The results obtained show mostly significantly positive correlations that indicate the growth of this crucial species of economic value (Fidalgo-e-Costa, Passos & Cancela-da-Fonseca, 2003; Gillet, 2018). We can therefore conclude that these structures evolve to accompany the maturation of the animal.

### Prostomial structures

From the results of the analysis of prostomial structures, such as the lateral antennae and palps, a significant positive correlation between their size and the number of chaetiger segments was revealed, indicating that when the number of chaetiger segments increases, these sensory structures also grow in width and length. The growth in the number of sensory appendages can be hypothesised as an expansion of sensory surfaces, which in turn leads to an enhancement of the sensory functions of these organs (this study). The increase of the sensory organs is coherent due to their high importance for the active lifestyle that these polychaetes have (Viéitez et al., 2004); in addition, these structures are believed to have a chemoreceptor role (Dorsett & Hyde, 1969). Besides that, when comparing the palp sections, a decrease in the number of collagen fibre layers in the palpostyles is a factor that could be significant in raising the sensitivity of the palpostyle (Almeida et al., 2025).

In this way, the continuous development of these sensory structures throughout the development of *H. diversicolor* suggests that, from the larval or juvenile stages to adulthood, this enhancement in perception of the surroundings helps the animal to navigate and interact more effectively with its environment, enabling more complex behaviours that are crucial for the survival and reproduction of the species (this study).

### Peristomial structures

The significant positive correlation between the number of paragnaths in most of the sections of the proboscis and the number of chaetiger segments shows that as the animal grows, the structures responsible for its feeding also undergo morphological adaptation, in this case, increasing the number of paragnaths. The paragnaths mainly help to grip the food particles and transfer them to the gut (Maltagliati et al., 2006). This increase probably positively influences the ability to capture a greater quantity of food, which is crucial since food requirements rise as the organism grows (this study). The length of the jaws, likewise, correlates positively with the number of chaetiger segments; however, the same is not true of the number of teeth in each jaw. The left and right jaws usually have the same number of teeth, and the number of teeth seems to be less variable than other characteristics. The number of teeth per jaw in *H. diversicolor*, between five and nine, is within the range of other nereidid species such as *Alitta succinea* (Leuckart, 1847) and other species of the genus *Hediste* (Hefferan, 1900; Sato & Nakashima, 2003). On the other hand, the results obtained for peristomial cirrus showed significant positive correlations between its measurements, including both cirrophore and cirrostyle, and the number of chaetiger segments. Thus, the growth of peristomial cirri is proportional to the growth of the organism. The peristomial cirri, like the palps and antennae of the prostomium, fulfil sensory functions. Therefore, it is expected that, as the worm ages, its sensory appendages become more voluminous, improving the reception of sensory stimuli, as was previously hypothesised in the case of the palps and antennae of the prostomium (this study).

### Parapodia structures and chaetae

The number of chaetiger segments and most of the measurements of the parapodial structures were positively correlated, also demonstrating a proportional growth pattern in which both the notopodium and the neuropodium grow as the organism develops new chaetiger segments. This same pattern was also observed in the nereidid species *Laeonereis acuta* (Treadwell, 1923), with the positive correlation between the increase in chaetigers and body size (Omena & Amaral, 2001). As the parapodia are key elements for the movement of these annelids (Hesselberg, 2006), as the size of the animal increases, there is a gradual adaptation of the structures that provide this function (this study). When the worm is younger and smaller, we hypothesise that parapodia with smaller dimensions and less developed will be able to perform the basic needs of locomotion and interactions with the sediment for its survival. As the annelid grows, its body gains more volume and mass, requiring its parapodia to become larger and more complex to maintain its ability to move, avoid predators and interact with the environment (Hesselberg, 2006; Hesselberg & Vincent, 2006; Meißner, Bick & Müller, 2012). Some parapodial structures, such as the notopodial cirrus of the anterior-median and median-posterior transitional parapodia, the neuropodial cirrus of the anterior-median, median and posterior median parapodia, the prL of the anterior-median, median-posterior and posterior parapodia, and poL and prL2 of the median-posterior transitional parapodia, did not show significant correlations. This means that although there is proportional growth of most of the parapodia’s structures, there are specific structures that grew at different rates, malformed or were influenced by other factors, such as environmental conditions, or mechanical wear/damage, depending on the location of the parapodium where they are found (this study). In the notopodium, there is no diversity in the type of chaetae, which are always of the homogomph spiniger type. In the neuropodium, there is diversity between the different parapodia along the body, particularly the transition from heterogomph falciger to simple chaetae, which appears when an individual of *H. diversicolor* reaches around 25 chaetiger segments (this study), undergoing a possible functional adaptation. For other species, of as *Hediste japonica* (Izuka, 1908) and other Asian species of *Hediste*, like *Hediste diadroma* Sato & Nakashima, 2003, parapodial development is conditioned by the life cycle phase (Sato & Nakashima, 2003; Kan et al., 2020). In these Asian species, the chaeta transitions occur in different body segments, depending on the stage of development and environmental variables (Sato, 2017), demonstrating greater flexibility in parapodial adaptation than in *H. diversicolor*. This distinction can be interpreted as an adaptation to the more variable habitats that *H. japonica* and *H. diadroma* inhabit, both being catadromous euryhaline nereidids that undergo epitokous metamorphosis (Sato, 2017; Kan et al., 2020), which requires a more adaptable locomotor apparatus. The presence of simple chaetae on the posterior segments of *H. diversicolor* may indicate a functional change as the worm matures, perhaps due to changes in interaction with the sediment or in burrowing behaviour as it approaches a mature state (this study).

### Relative position of the first parapodium with simple chaetae

The presence of simple chaetae on the posterior segments of *H. diversicolor* may indicate a functional change as the worm matures. Statistical analysis on the relative position of the first parapodium with simple chaeta in relation to the number of chaetiger segments revealed a significant negative correlation, indicating that during annelid development, simple chaetae occur earlier along the body axis. This ontogenetic shift in parapodial chaeta could be linked to the worm’s transition from juvenile to adult stages when modifications in burrowing behaviour or substrate interaction become necessary due to their enlarged size (this study). Simple chaetae are thicker and stronger, possibly being used to better anchor their bodies to the burrows as a defence mechanism (Hesselberg & Vincent, 2006), and potentially help in reproductive activities, since in this species fertilisation takes place inside the burrow (Scaps, 2002). This growth tendency is consistent with observations in other *Hediste* species, such as *H. japonica* and *H. atoka*, possibly demonstrating a synapomorphic condition (this study), where the first chaetiger with simple chaetae emergence is similar to the data obtained from *H. diversicolor* (Sato & Nakashima, 2003). For individuals with approximately 100 chaetiger segments, the first parapodia with simple chaetae was discovered to be near the 50th chaetiger segment.

**Table 1:** Individual growth rates in length and width of each studied structure from the prostomium per each new chaetiger segment.

		Individual growth rates growth rates per each new chaetiger	
	Structure	Length	Width
	Lateral Antennae	0.0047	0.0017
**Palp**	Left Palpophore	0.0096	0.0090
	Right Palpophore	0.0097	0.0089
	Left Palpostyle	0.0016	0.0014
	Right Palpostyle	0.0026	0.0024

**Table 2:** Individual growth rates in length and width of each studied structure from the peristomium per each new chaetiger segment.

		Individual growth rates per each new chaetiger	
	Structure	Length	Width
	Jaw	0.0127	–
**Cirri 1**	Left Cirrophore	0.0024	0.0072
	Right Cirrophore	0.0025	0.0071
	Left Cirrostyle	0.0086	0.0018
	Right Cirrostyle	0.0084	0.0017
**Cirri 2**	Left Cirrophore	0.0027	0.0027
	Right Cirrophore	0.0033	0.0029
	Left Cirrostyle	0.0205	0.0017
	Right Cirrostyle	0.017	0.0018
**Cirri 3**	Left Cirrophore	0.0021	0.0023
	Right Cirrophore	0.0023	0.0022
	Left Cirrostyle	0.0159	0.0016
	Right Cirrostyle	0.0133	0.0017
**Cirri 4**	Left Cirrophore	0.0038	0.0027
	Right Cirrophore	0.0042	0.0029
	Left Cirrostyle	0.0292	0.0019
	Right Cirrostyle	0.0272	0.0017

**Table 3:** Individual growth rates in length and width of each studied structure of parapodia from different body sections per each new chaetiger segment.

			Individual growth rates per each new chaetiger	
		Structure	Length	Width
**Anterior**	**Noto.**	Noto. Cirri	0.0054	0.0013
		noL1	0.0080	0.0037
	**Neuro.**	Neuro. Cirri	0.0057	0.0013
		prL2	0.0073	0.0022
		poL	0.0078	0.0021
		neL	0.0074	0.0029
		Acicula	0.0108	–
**Transition Anterior-Median**	**Noto.**	Noto. Cirri	0.0034	0.0007
		noL1	0.0205	0.0065
		prL	0.0040	0.0018
		noL2	0.0190	0.0032
		Acicula	0.0170	–
	**Neuro.**	Neuro. Cirri	0.0023	0.0009
		prL2	0.0181	0.0033
		poL	0.0197	0.0022
		neL	0.0147	0.0020
		Acicula	0.0190	–
**Before Simple chaetae**	**Noto.**	Noto. Cirri	0.0038	0.0011
		noL1	0.0233	0.0059
		prL	0.0045	0.0021
		noL2	0.0182	0.0030
		Acicula	0.0254	–
	**Neuro.**	Neuro. Cirri	0.0035	0.0016
		prL2	0.0154	0.0027
		poL	0.0150	0.0029
		neL	0.0142	0.0017
		Acicula	0.0235	–
**First With Simple Chaetae**	**Noto.**	Noto. Cirri	0.0040	0.0010
		noL1	0.0225	0.0047
		prL	0.0049	0.0017
		noL2	0.0177	0.0027
		Acicula	0.0255	–
	**Neuro.**	Neuro. Cirri	0.0036	0.0009
		prL2	0.0154	0.0016
		poL	0.0149	0.0015
		neL	0.0115	0.0017
		Acicula	0.0253	–
**Median**	**Noto.**	Noto. Cirri	0.0036	0.0009
		noL1	0.0180	0.0053
		prL	0.0046	0.0016
		noL2	0.0163	0.0026
		Acicula	0.0235	–
	**Neuro.**	Neuro. Cirri	0.0018	0.0008
		prL2	0.0128	0.0018
		poL	0.0124	0.0023
		neL	0.0089	0.0016
		Acicula	0.0226	–
**Transition Median-Posterior**	**Noto.**	Noto. Cirri	0.0017	0.0006
		noL1	0.0128	0.0029
		prL	0.0006	0.0003
		noL2	0.0103	0.0016
		Acicula	0.0163	–
	**Neuro.**	Neuro. Cirri	0.0013	0.0008
		prL2	0.0080	0.0002
		poL	0.0077	0.0004
		neL	0.0064	0.0007
		Acicula	0.0146	–
**Posterior**	**Noto.**	Noto. Cirri	0.0045	0.0009
		noL1	0.0116	0.0020
		prL	0.0015	0.0004
		noL2	0.0106	0.0015
		Acicula	0.0123	–
	**Neuro.**	Neuro. Cirri	0.0025	0.0005
		prL2	0.0075	0.0008
		poL	0.0076	0.0010
		neL	0.0072	0.0010
		Acicula	0.0123	–

**Table 4:** Individual growth rates in length of the pygidial cirri and cirri segments per each new chaetiger segment.

		Individual growth rates per each new chaetiger
	Structure	Length
**Pygidium**	Left Pygidial Cirrus	0.0372
	Right Pygidial Cirrus	0.0346
	Cirri Segment	0.0022

### Pygidial cirri

The significative positive correlation results obtained between the length of the pygidial cirri, their cirrus segments and the chaetiger number in *H. diversicolor*, again show that these structures developing in proportion to the rest of the body, further reinforcing the idea that the ragworm grows uniformly in each region of the body, adjusting the dimensions of each structure to maintain the corresponding functionality as the organism increases in size (this study). The pygidial cirri, having sensory function (Starunov, 2019), follow the same trend as the other sensory appendages of the prostomium and peristomium.

## Conclusions

This is the first biometric study using chaetigers segments as a primary parameter for analysing annelid growth, leading to gradual modifications in the body and its appendages and structures. From the results obtained in the correlation analyses between the various structures with different functions, such as sensory, locomotor and feeding, in relation to the number of chaetiger segments, an indicator of growth, important information was discovered about the growth patterns and morphological adaptations of *H. diversicolor*, illustrating how its body develops throughout growth, and demonstrating the complex developmental processes that shape the anatomy of this species of nereid, differentiating it from other species in the genus *Hediste*. For example, the increase in the quantity of sensory appendages may be theorised as an enlargement of sensory surfaces, which subsequently results in an improvement of the sensory capabilities of prostomial characters.

It should be noted that there is a proportional growth trend between most of the structures analysed and the overall growth of the body, and that in the case of some specific structures, they can be subject to different selective pressures or developmental restrictions. We detected that simple chaetae arising from individuals with 25 chaetiger segments, possibly indicating a transitional stage towards maturation. These results show the evolutionary adaptability of the genus *Hediste* and contribute to our understanding of the morphological variability of polychaetes. They can also guide future research into specific characteristics of the *H. diversicolor* that were previously unknown, given that animal growth and development tracking of certain key features was lacking. Finally, this new biometric methodology can be applied to all annelids.

##  Supplemental Information

10.7717/peerj.20736/supp-1 Supplemental Information 1 Loadings of Principal Component Analysis for Multivariate Analyses (palps, paragnaths, peristomial cirri, anterior parapodia and median-posterior parapodia)

10.7717/peerj.20736/supp-2 Supplemental Information 2 Raw data

## Additional Information and Declarations

### Data Availability

The following information was supplied regarding data availability: The biometric and morphological data (raw data) are available in the Supplemental Information.
