# Supplementary material for: Establishing a new methodology for annelid studies: a biometric study of the ragworm Hediste diversicolor (Müller, 1776)
Source: PeerJ. 2026 Feb 3;14:e20736. doi: 10.7717/peerj.20736 (PMC12880096; doi:10.7717/peerj.20736)
Supplement: Supplemental Information 1 [file peerj-14-20736-s001.docx]

Supplementary Material

**Table S1.** Loadings of Principal Component Analysis for Multivariate Analyses (palps, paragnaths, peristomial cirri, anterior parapodia and median-posterior parapodia).

PCA palps

| Character | Loading PC1 |
| --- | --- |
| P_phore_left_length | -0.51 |
| P_phore_left_width | -0.48 |
| P_phore_rigth_length | -0.50 |
| P_phore_right_width | -0.47 |
| P_style_left_length | -0.07 |
| P_style_left_width | -0.13 |
| P_style_rigth_length | -0.06 |
| P_style_right_width | -0.12 |

PCA proboscis paragnaths

| Character | Loading PC1 |
| --- | --- |
| I | -0.06 |
| IIa | -0.16 |
| Iib | -0.14 |
| III | -0.63 |
| IVa | -0.45 |
| IVb | -0.45 |
| VIa | -0.03 |
| VIb | -0.02 |
| VII.VIII | -0.38 |

PCA peristomial cirri

| Character | Loading PC1 |
| --- | --- |
| X1_phore_left_lenght | 0.04 |
| X1_phore_left_width | 0.04 |
| X1_phore_rigth_lenght | 0.04 |
| X1_phore_right_width | 0.04 |
| X1_style_left_lenght | 0.15 |
| X1_style_left_width | 0.03 |
| X1_styles_rigth_lenght | 0.15 |
| X1_style_right_width | 0.03 |
| X2_phore_left_lenght | 0.05 |
| X2_phore_left_width | 0.04 |
| X2_phore_rigth_lenght | 0.06 |
| X2_phore_right_width | 0.04 |
| X2_style_left_lenght | 0.38 |
| X2_style_left_width | 0.03 |
| X2_styles_rigth_lenght | 0.31 |
| X2_style_right_width | 0.03 |
| X3_phore_left_lenght | 0.04 |
| X3_phore_left_width | 0.04 |
| X3_phore_rigth_lenght | 0.04 |
| X3_phore_right_width | 0.04 |
| X3_style_left_lenght | 0.25 |
| X3_style_left_width | 0.03 |
| X3_styles_rigth_lenght | 0.21 |
| X3_style_right_width | 0.03 |
| X4_phore_left_lenght | 0.07 |
| X4_phore_left_width | 0.04 |
| X4_phore_rigth_lenght | 0.07 |
| X4_phore_right_width | 0.04 |
| X4_style_left_lenght | 0.53 |
| X4_style_left_width | 0.03 |
| X4_styles_rigth_lenght | 0.54 |
| X4_style_right_width | 0.03 |

PCA anterior parapodia

| Character | Loading PC1 |
| --- | --- |
| A_Cirri_length | -0.05 |
| A_Cirr_width | -0.01 |
| A_Nol1_length | -0.08 |
| A_Nol1_width | -0.04 |
| B_Cirri_length | -0.05 |
| B_Cirri_width | -0.01 |
| B_rL2_length | -0.07 |
| B_rL2_width | -0.02 |
| B_poL_length | -0.10 |
| B_poL_width | -0.03 |
| B_neL_length | -0.08 |
| B_neL_width | -0.03 |
| B_articula_length | -0.11 |
| C_Cirri_length | -0.03 |
| C_Cirri_with | -0.01 |
| C_noL1_length | -0.23 |
| C_noL1_width | -0.07 |
| C_prL_length | -0.05 |
| C_prL_width | -0.02 |
| C_noL2_length | -0.21 |
| C_noL2_width | -0.03 |
| C_articula_length | -0.18 |
| D_Cirri_length | -0.02 |
| D_Cirri_width | -0.01 |
| D_rL2_length | -0.20 |
| D_rL2_width | -0.03 |
| D_poL_length | -0.21 |
| D_poL_width | -0.02 |
| D_neL_length | -0.16 |
| D_neL_width | -0.02 |
| D_articula_length | -0.20 |
| E_Cirri_length | -0.02 |
| E_Cirri_with | -0.01 |
| E_noL1_length | -0.27 |
| E_noL1_width | -0.06 |
| E_prL_length | -0.04 |
| E_prL_width | -0.02 |
| E_noL2_length | -0.20 |
| E_noL2_width | -0.03 |
| E_articula_length | -0.27 |
| F_Cirri_length | -0.02 |
| F_Cirri_width | -0.02 |
| F_rL2_length | -0.17 |
| F_rL2_width | -0.03 |
| F_poL_length | -0.16 |
| F_poL_width | -0.03 |
| F_neL_length | -0.17 |
| F_neL_width | -0.01 |
| F_articula_length | -0.25 |
| G_Cirri_length | -0.04 |
| G_Cirri_with | -0.01 |
| G_noL1_length | -0.25 |
| G_noL1_width | -0.04 |
| G_prL_length | -0.04 |
| G_prL_width | -0.01 |
| G_noL2_length | -0.20 |
| G_noL2_width | -0.02 |
| G_articula_length | -0.26 |
| H_Cirri_length | -0.03 |
| H_Cirri_width | -0.01 |
| H_rL2_length | -0.16 |
| H_rL2_width | -0.01 |
| H_poL_length | -0.16 |
| H_poL_width | -0.01 |
| H_neL_length | -0.12 |
| H_neL_width | -0.01 |
| H_articula_length | -0.27 |

PCA median-posterior parapodia

| Character | Loading PC1 |
| --- | --- |
| AM_Cirri_length | -0.03 |
| AM_Cirri_with | -0.01 |
| AM_noL1_length | -0.29 |
| AM_noL1_width | -0.08 |
| AM_prL_length | -0.05 |
| AM_prL_width | -0.02 |
| AM_noL2_length | -0.25 |
| AM_noL2_width | -0.04 |
| AM_articula_length | -0.36 |
| BM_Cirri_length | -0.02 |
| BM_Cirri_width | -0.01 |
| BM_rL2_length | -0.21 |
| BM_rL2_width | -0.03 |
| BM_poL_length | -0.20 |
| BM_poL_width | -0.04 |
| BM_neL_length | -0.14 |
| BM_neL_width | -0.02 |
| BM_articula_length | -0.35 |
| CM_Cirri_length | -0.04 |
| CM_Cirri_with | -0.01 |
| CM_noL1_length | -0.19 |
| CM_noL1_width | -0.05 |
| CM_prL_length | -0.03 |
| CM_prL_width | -0.01 |
| CM_noL2_length | -0.16 |
| CM_noL2_width | -0.03 |
| CM_articula_length | -0.28 |
| DM_Cirri_length | -0.02 |
| DM_Cirri_width | -0.01 |
| DM_rL2_length | -0.12 |
| DM_rL2_width | -0.01 |
| DM_poL_length | -0.11 |
| DM_poL_width | -0.01 |
| DM_neL_length | -0.09 |
| DM_neL_width | -0.01 |
| DM_articula_length | -0.23 |
| EM_Cirri_length | -0.06 |
| EM_Cirri_with | -0.01 |
| EM_noL1_length | -0.23 |
| EM_noL1_width | -0.04 |
| EM_prL_length | -0.02 |
| EM_prL_width | -0.01 |
| EM_noL2_length | -0.20 |
| EM_noL2_width | -0.02 |
| EM_articula_length | -0.22 |
| EM_Cirri_length.1 | -0.03 |
| EM_Cirri_width | -0.01 |
| EM_rL2_length | -0.14 |
| EM_rL2_width | -0.01 |
| EM_poL_length | -0.14 |
| EM_poL_width | -0.01 |
| EM_neL_length | -0.13 |
| EM_neL_width | -0.02 |
| EM_articula_length.1 | -0.21 |
