# Supplementary material for: Establishing a new methodology for annelid studies: a biometric study of the ragworm Hediste diversicolor (Müller, 1776)
Source: PeerJ. 2026 Feb 3;14:e20736. doi: 10.7717/peerj.20736 (PMC12880096; doi:10.7717/peerj.20736)
Supplement: Supplemental Information 2 [file peerj-14-20736-s002.docx]

# **Supplementary Material (Tables S2-S7)**

**Table S2.** Biometric and morphological data on different structures of the proboscis and prostomium of *Hediste diversicolor*.

**Table S3.** Biometric and morphological data on different structures of the peristomium of *Hediste diversicolor*.

**Table S4.** Biometric and morphological data on different structures of *Hediste diversicolor* from the anterior parapodium and the anterior-median transitional parapodium.

**Table S5.** Biometric and morphological data on different structures of *Hediste diversicolor* from the parapodium before the first parapodium with simple chaeta, and from the first parapodium with simple chaeta.

**Table S6.** Biometric and morphological data on different structures of *Hediste diversicolor* from the median parapodium and the median-posterior transitional parapodium.

**Table S7.** Biometric and morphological data on different structures of *Hediste diversicolor* from the posterior parapodium and pygidium.
